
JOURNAL INFORMATION
==============================
NLM Title Abbreviation: ScientificWorldJournal
Journal ID: ScientificWorldJournal
NLM Journal ID: 101131163
Journal ID: TSWJ

The Scientific World Journal

ISSN: 2356-6140
EISSN: 1537-744X
Publisher: Wiley

ARTICLE INFORMATION
==============================
PMCID: PMC5763827
PMID: 20062953
DOI: 10.1100/tsw.2010.6
Article ID: 652974
Article version: 1
Subjects: Clinical Image

A Giant Inguinoscrotal Hernia

Chernev Ivan *
Department of Rehabilitation Medicine, Boston University Medical Center, Boston, MA, USA
*Ivan Chernev: ivantchernev@yahoo.com, ivan.chernev@bmc.orgAcademic Editor: Daniel Batlle

Publication date: 2010
Electronic publication date: 2010 Jan 8
Volume: 10
First page: 72
Last page: 73
Received 2009 Nov 5; Revised 2009 Dec 6; Accepted 2009 Dec 11
Copyright: Copyright © 2010 Ivan Chernev.
Copyright year: 2010
License: This is an open access article distributed under the Creative Commons Attribution License, which permits unrestricted use, distribution, and reproduction in any medium, provided the original work is properly cited.
License URL: https://creativecommons.org/licenses/by/3.0/

Keywords: giant inguinoscrotal hernia

==============================
Clinical Image
*TheScientificWorldJOURNAL* (2010) 10, 72–73
TSW Clinical Images
ISSN 1537-744X; DOI 10.1100/tsw.2010.6

TheScientificWorldJOURNAL
www.thescientificworld.com

### A Giant Inguinoscrotal Hernia

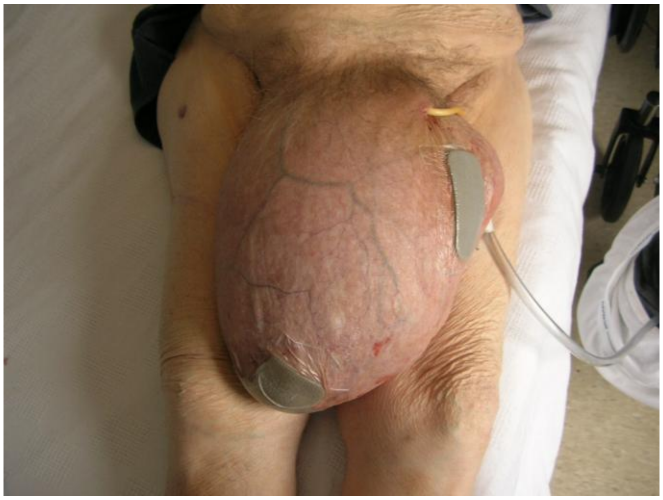

**FIGURE.** A giant inguinoscrotal hernia extending to the knee level.

Ivan Chernev
*Department of Rehabilitation Medicine, Boston University Medical Center, Boston, MA*

**E-mail**: ivantchernev@yahoo.com or Ivan.Chernev@bmc.org

*Received November 5, 2009; Revised December 6, 2009; Accepted December 11, 2009; Published January 8, 2010*

**KEYWORDS:** giant inguinoscrotal hernia

Giant inguinoscrotal hernias are uncommon nowadays, however, they may still occasionally present after years of neglect[1]. Significant morbidity and decreased quality of life may be associated with them. They may cause urinary retention, skin ulcers and infection, and decreased mobility apart from the classical complications of inguinoscrotal hernias[2].

A 71-year-old man with multiple medical problems, including chronic cardiac and pulmonary disease, was admitted to our inpatient rehabilitation unit after acute hospitalization due to pneumonia. During the routine physical examination, a huge inguinoscrotal hernia extending to the knee level was observed (see figure). The patient reported that he noticed the hernia 7 years ago and it has been increasing in size since then. His mobility was decreased due to the size of the hernia as well as general deconditioning. After discussion with the patient, the decision was made not to proceed with surgical treatment due to the significantly increased operative risk.

Although surgical treatment seems to be the only reasonable treatment for giant inguinoscrotal hernias, forced reduction of hernial contents back into the contracted peritoneal cavity may alter the intra-

©2009 with author.

abdominal and intrathoracic pressures, leading to added morbidity and mortality. Several single- or multiple-stage surgical techniques have been described. These techniques include three different groups of surgeries. The first group consists of debulking the abdominal contents, i.e., omentectomy, bowel resection, and splenectomy[1,3]. The second group includes different procedures for enlarging the abdominal cavity, such as preoperative pneumoperitoneum, skin and muscle flaps, mesh patches, and phrenectomy[4,5]. The last group uses a combination of techniques from the first two groups. Regardless of the selected surgical management, the morbidity associated with giant inguinoscrotal hernias can be significant, with most of the cases reporting minor or major postoperative complications. Unfortunately, patients with giant inguinoscrotal hernias frequently have comorbid conditions, such as cardiac and pulmonary disease, which may complicate the surgery even further. In patients with multiple medical conditions, adequate preoperative preparation and postoperative monitoring is imperative in case surgical treatment is opted.

### REFERENCES

1. Patsas, A., Tsiaousis, P., Papaziogas, B., Koutelidakis, I., Goula, C., and Atmatzidis, K. (2009) Repair of a giant inguinoscrotal hernia. *Hernia*. [Epub ahead of print]
2. Vano-Galvan, S., Guisado-Vasco, P., and Jaén, P. (2009) Giant inguinoscrotal hernia. *Aust. Fam. Physician* **38(4),** 222–223.
3. El Saadi, A.S., Al Wadan, A.H., and Hamerna, S. (2005) Approach to a giant inguinoscrotal hernia. *Hernia* **9(3),** 277–279.
4. Kovachev, L.S., Paul, A.P., Chowdhary, P., Choudhary, P., and Filipov, E.T. (2009) Regarding extremely large inguinal hernias with a contribution of two cases. *Hernia.* [Epub ahead of print]
5. Valliattu, A.J. and Kingsnorth, A.N. (2008) Single-stage repair of giant inguinoscrotal hernias using the abdominal wall component separation technique. *Hernia* **12(3),** 329–330.

**This article should be cited as follows:**

Chernev, I. (2010) A giant inguinoscrotal hernia. *TheScientificWorldJOURNAL*: TSW Clinical Images **10**, 72–73. DOI 10.1100/tsw.2010.6.
